# Supplementary material for: Complexome profiling of the Chlamydomonas psb28 mutant reveals TEF5 as an early PSII assembly factor
Source: Plant Cell. 2025 Mar 17;37(6):koaf055. doi: 10.1093/plcell/koaf055 (PMC12164590; doi:10.1093/plcell/koaf055)
Supplement: koaf055_Supplementary_Data [file koaf055_supplementary_data.zip › psb28_interactive.zip!/plots/intensity/qualityPlots/peptides/_Cre10.g453000.t1.2.html]

### Cre10.g453000.t1.2

Trivial name:   
SeqCoverage [median]: 17.80  
Mass: 24.89 kDa  
  
 

| Sequence | MissedCleavage | Mass [Da] | wtIntensity | muIntensity | Score |
| --- | --- | --- | --- | --- | --- |
| SNVNVLENK | 0 | 1015.53 | 293.61 | 0.00 | 81.95 |
| SNVNVLENKK | 1 | 1143.62 | 1077.68 | 2541.01 | 81.77 |
|  |  |  | 1371.3 | 2541.0 |  |
